# Comparing the performance of machine learning and conventional models for predicting atherosclerotic cardiovascular disease in a general Chinese population

**DOI:** 10.1186/s12911-023-02242-z

**Published:** 2023-07-24

**Authors:** Zihao Fan, Zhi Du, Jinrong Fu, Ying Zhou, Pengyu Zhang, Chuning Shi, Yingxian Sun

**Affiliations:** 1 Department of Cardiology, The First Hospital of China Medical University, No. 155, Nanjing Bei Street, Shenyang, 110001 China; 2 Guangdong Provincial People’s Hospital (Guangdong Academy of Medical Sciences), Southern Medical University, 106 Zhongshan 2nd Road, Guangzhou, 510080 China; 3 Department of Cardiology, The First Affiliated Hospital, Zhejiang University School of Medicine, Hangzhou, China; 4 Department of Endocrinology and Metabolism, The First Hospital of China Medical University, No. 155, Nanjing Bei Street, Shenyang, 110001 China

**Keywords:** Machine learning, Risk Assessment/classification, Electrocardiography, Echocardiography, ASCVD

## Abstract

**Background:**

Accurately predicting the risk of atherosclerotic cardiovascular disease (ASCVD) is crucial for implementing individualized prevention strategies and improving patient outcomes. Our objective is to develop machine learning (ML)-based models for predicting ASCVD risk in a prospective Chinese population and compare their performance with conventional regression models.

**Methods:**

A hybrid dataset consisting of 551 features was used, including 98 demographic, behavioral, and psychological features, 444 Electrocardiograph (ECG) features, and 9 Echocardiography (Echo) features. Seven machine learning (ML)-based models were trained, validated, and tested after selecting the 30 most informative features. We compared the discrimination, calibration, net benefit, and net reclassification improvement (NRI) of the ML models with those of conventional ASCVD risk calculators, such as the Pooled Cohort Equations (PCE) and Prediction for ASCVD Risk in China (China-PAR).

**Results:**

The study included 9,609 participants (mean age 53.4 ± 10.4 years, 53.7% female), and during a median follow-up of 4.7 years, 431 (4.5%) participants developed ASCVD. In the testing set, the final ML-based ANN model outperformed PCE, China-PAR, recalibrated PCE, and recalibrated China-PAR in predicting ASCVD. This was demonstrated by the model's higher area under the curve (AUC) of 0.800, compared to 0.777, 0.780, 0.779, and 0.779 for the other models, respectively. Additionally, the model had a lower Hosmer–Lemeshow χ2 of 9.1, compared to 37.3, 67.6, 126.6, and 18.6 for the other models. The net benefit at a threshold of 5% was also higher for the ML-based ANN model at 0.017, compared to 0.016, 0.013, 0.017, and 0.016 for the other models, respectively. Furthermore, the NRI was 0.089 for the ML-based ANN model, while it was 0.355, 0.098, and 0.088 for PCE, China-PAR, and recalibrated PCE, respectively.

**Conclusions:**

Compared to conventional regression ASCVD risk calculators, such as PCE and China-PAR, the ANN prediction model may help optimize identification of individuals at heightened cardiovascular risk by flexibly incorporating a wider range of potential predictors. The findings may help guide clinical decision-making and ultimately contribute to ASCVD prevention and management.

**Supplementary Information:**

The online version contains supplementary material available at 10.1186/s12911-023-02242-z.

## Introduction

Atherosclerotic cardiovascular disease (ASCVD), defined as nonfatal acute myocardial infarction, coronary heart disease (CHD) death, and stroke, has become the leading cause of morbidity and mortality worldwide [1]. In China, cardiovascular disease (CVD) accounts for 38.9% of deaths among females and 35.5% of deaths among males [2], and approximately 61% of these deaths were attributed to ASCVD [3]. Identification of high-risk individuals is critical for the primary prevention of ASCVD. However, personalized assessment of cardiovascular risk remains a challenge in clinical practice. Current medical guidelines recommend the use of risk calculator to perform risk assessment and guide decision-making in ASCVD management [4–7]. The 2017 American College of Cardiology/American Heart Association (ACC/AHA) guideline highlighted the use of the pooled cohort equations (PCE) to determine 10-year ASCVD risk [5], which present a widely used guideline-endorsed risk calculator in clinical setting. The PCE were derived primarily from non-Hispanic Whites and African-American populations, over- and underestimation of risk have been reported for specific population cohorts [8–11]. Recently, the Prediction for ASCVD Risk in China (China-PAR) equations were published as a risk prediction calculator designed for Chinese adults and has been approved by the Chinese guidelines in 2019 [7], while more evidence is warranted to confirm its generality for different population.

Notably, recent evidence suggested that besides traditional risk factors of ASCVD (age, sex, smoking, etc.), parameters from electrocardiography (ECG) and echocardiography (Echo) are also helpful in predicting the risk of CVD by detection of subclinical impairment before cardiac symptoms appear [12]. Abnormality in P wave, PR intervals, and left ventricular ejection fraction (LVEF) has been demonstrated to be associated with adverse cardiovascular outcomes [12–17]. However, these massive amounts of parameters reflecting the electrophysiology, structure, and function of the heart were unlikely to be included in traditional prediction models. In this context, developing a trainable model for different populations may provide novel insights for the prevention of ASCVD.

Machine learning (ML) offers a novel approach for individualized risk assessment, which could incorporate variables that may not be considered in traditional regression algorithms to develop risk prediction model by exploring the multidimensional and nonlinear relationship between potential variables [18]. Many studies have applied machine learning to the evaluation of various disease and with good results [19–21]. Although there has been a rapid expansion of ML being applied to cardiology [22–24], few direct comparisons have been made between ML and traditional ASCVD models [25, 26], none of these studies have included the Chinese population.

The present study aimed to establish ML-based risk prediction models from the dataset that integrates demographic, behavioral, psychological, Electrocardiograph and Echocardiography variables to predict ASCVD in a community-based general population in Northeast China. Meanwhile, we compared the performance of ML algorithms to traditional Cox regression models (PCE and China-PAR) to evaluate which method provided superior predictive performance.

## Methods

### Study population

The Northeast China Rural Cardiovascular Health Study (NCRCHS) is a multistage, stratified, random cluster sampling prospective population cohort of 11,956 participants aged ≥ 35 years, recruited between Jan 9, 2013, to Aug 23, 2013, from rural residents living in the Liaoning Province, China. Demographics, physical status and vitals, medical histories, echocardiography data, ECG exams, and laboratory data were collected. Consistent with the target population of contemporary risk prediction scores, participants were included in case of age between 35 and 85 years, no history of CVD. Of 11,956 participants assessed for eligibility, 10,349 (86.6%) participants completed at least one follow-up visit.

### Clinical demographics

At baseline face-to-face interviews, detailed information included Clinical demographics (sex, age, marriage, education, nation, etc.) as well as lifestyle factors and (family) medical histories (heart disease, stroke, diabetes, hypertension, etc.) were collected using standardized questionnaires by trained staff (Supplementary file 1). Weight (the nearest 0.1 kg), height and waist circumference (the nearest 0.1 cm) of participants were measured. The body mass index (BMI) was calculated as weight in kilograms divided by height in meters squared. Blood pressure (BP) was measured using a standardized automatic electronic sphygmomanometer (HEM-907; Omron, Tokyo, Japan) after 5 min of rest for three times, and the mean values of systolic/diastolic blood pressure were calculated. The questionnaire was checked by trained staff at the end of each participant’s follow-up to ensure that the data collected were complete and accurate. The paper questionnaire was manually double-entered and subsequently saved. Blood samples from all participants were collected in the morning after > 12 h of overnight fasting.

Hypertension was defined as a mean systolic blood pressure > 140 mmHg and/or diastolic blood pressure > 90 mmHg or taking antihypertensive medications. Diabetes mellitus was defined by medical history and/or use of insulin or oral hypoglycemic agents. Participants were considered to be current smokers/drinkers if they had smoked/drank at any point in the 3 months prior to the date of the ECG examination.

### Electrocardiograph and Echocardiography measurement

Standard 12-lead ECGs (MAC 5500, GE Healthcare, Little Chalfont, UK) were recorded in the resting supine position at baseline and were analyzed automatically with the MUSE Cardiology Information System, version 7.0.0 (GE Marquette™ 12SL™ ECG analysis program) [27]. Total of 645 parameters from the unprocessed digital ECG data were disposed by the GE system. 201 parameters (9 not lead-specific and 192[16*12] lead-specific) were temporally stored that including the relative coordinate points (the start point of the p-wave, etc.), and calculated values (QTc Framingham and QTc Fridercia, etc.) were excluded. The remaining 444 variables were used for analysis.

Transthoracic doppler echocardiography (Vivid; GE Healthcare, Connecticut, USA) constituted M-mode, two-dimensional, spectral, and color Doppler formats were operated by the sonographers with a 3.0-MHz transducer. Three professional doctors performed readings and analysis of the echocardiogram and had the option to consult two additional specialists if questions or uncertainties arose. Total of 9 echocardiographic parameters were used for analysis.

### Outcome assessment

Primary endpoints include stroke and CHD. Health status, hospital admissions, outpatient diagnosis, and deaths of each participant were followed up from 2015 to 2018. Two physicians reviewed medical records independently, categorized the events and specified the event dates. Stroke was defined as a sudden onset of focal neurological dysfunction lasting 24 h or until death, or less than 24 h but with a clinically relevant brain lesion. CHD was defined to include any myocardial infarction (MI), resuscitated cardiac arrest, definite angina, probable angina followed by revascularization, and CHD death.

### Machine learning

#### Feature selection

In this study, a total of 635 candidate variables were collected and 84 variables with a missing ratio greater than 10% were excluded. Missing values were imputed using the Multiple imputation method when missingness was < 10%. The filtered dataset included 551 variables: 98 demographics, behavioral and psychological variables (age, sex, BP, BMI, lifestyle, biochemical test, etc.), 444 ECG parameters and 9 Echo parameters. The detailed descriptions of features are available in Table S1. Feature selection was implemented using an approach known as ‘Recursive Feature Elimination’ (RFE) to reduce the feature dimension and find out the most discriminative information by selecting the most relevant variables and removing redundant variables. During the recursion process, an optimal subset of candidates is generated by eliminating the least important features from the complete feature set (Figure S1).

### Feature importance

To determine the major predictors of ASCVD in our study population, the importance of each permutation feature was judged from the final model. Permutation feature importance weighs the importance of each feature by calculating the increase in the prediction error of the model after permuting its values. A feature is considered important if removing its values decreases the discriminative capability of the model, as the model depends significantly on that feature for prediction. A feature is immaterial if removing its values but the mean area under the receiver operating characteristic curve (AUC) remains the same, as the model ignores the feature for prediction in this case.

### Model building and testing

The datasets were randomized into training (80%) and testing sets (20%). Model development included trials of several ML classifiers such as Artificial Neural Network (ANN), Random Forest (RF), Gradient Boosting Machine (GBM), K Nearest Neighbors (KNN), Adaptive Boosting (AdaBoost), Support Vector Machine (SVM), Categorical Boosting (CatBoost). Models were trained using optimal subset and evaluated with stratified 10-fold cross-validation on the training set and we used a grid search approach to determine the appropriate hyperparameters of each ML model [9, 28] (Table S2). To solve the class imbalance in the datasets, we assigned more weights to the minority class sample to increase the misclassification cost of minority class samples. We then evaluated the performance of PCE (White), China-PAR, Recalibrated PCE (White), Recalibrated China-PAR and ML-based risk prediction model in terms of discrimination, calibration, net benefit, and net reclassification improvement (NRI) (Table S3).

### Statistical analysis

Categorical variables are presented as count (%), and continuous variables are reported as mean (± SD). Brier score and Matthews correlation coefficient (MCC) were used to assessing the overall performance [29, 30]. The calibration of the models was tested with Hosmer–Lemeshow χ2 statistic [31]. Pairwise comparisons were performed between all predictive models using the DeLong test [32]. Decision-curve analysis (DCA) was used to quantify the net benefit of each risk prediction model [33]. Statistical significance was defined as two-tailed *P* < 0.05. All analyses were performed with R version 4.1.2.

## Results

### Study population

**Fig. 1:**
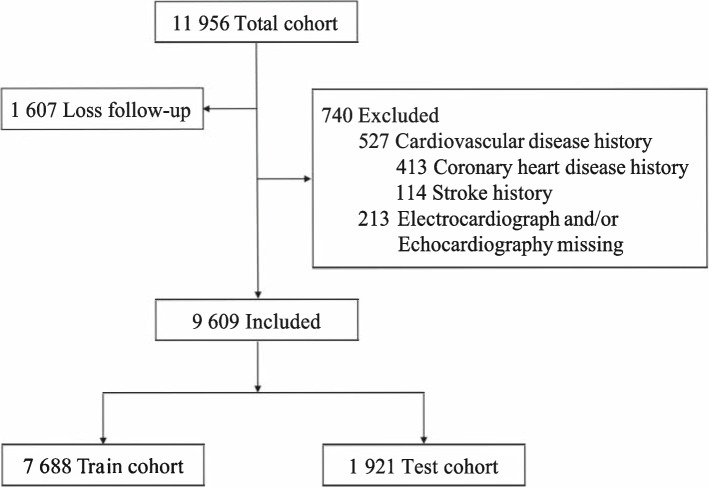
Flow chart of inclusion of participants for final analyses

A total of 9,609 participants (mean [SD] age: 53.4 [10.4] years; male [46.3%]) with digital ECG and Echo free of CVDs at baseline were included in the final cohort **(**Fig. 1). During a median of 4.7 (IQR, 4.4–4.9) years, 431 (4.5%) participants developed ASCVD (Table 1). 7,688 participants were included in the training cohort and 1,921 in the test cohort.

**Table 1:** Baseline clinical characteristics

**Characteristics**	**Total** **(** ***n*** ** = 9609)**	**ASCVD** **(** ***n*** ** = 431)**
Age at enrollment (year) [mean, ± SD]	53.4 (± 10.4)	62.4 (± 9.2)
Male gender (%)	4453 (46.3%)	244 (56.6%)
BMI (kg/m^2^) [mean, ± SD]	24.8 ± 3.7	25.1 ± 3.6
SBP (mmHg) [mean, ± SD]	141.8 ± 23.3	160.2 ± 27.6
DBP (mmHg) [mean, ± SD]	82.1 ± 11.7	87.6 ± 14.6
GLU (mmol/L) [mean, ± SD]	5.9 ± 1.6	6.22 ± 1.9
eGFR (mL/min/1.73m2) [mean, ± SD]	94.0 ± 15.1	84.7 ± 15.2
CR (μmol/L) [mean, ± SD]	71.1 ± 18.3	77.6 ± 39.5
WBC (10^9^/L) [mean, ± SD]	6.2 ± 1.9	6.3 ± 1.9
UA (μmol/L) [mean, ± SD]	287.5 ± 82.7	306.8 ± 88.0
BUN (mmol/L) [mean, ± SD]	5.6 ± 2.2	5.9 ± 1.7
AST (U/L) [mean, ± SD]	22.3 ± 12.0	24.0 ± 15.3
LDL (mmol/L) [mean, ± SD]	2.9 ± 0.8	3.2 ± 0.9
HDL (mmol/L) [mean, ± SD]	1.4 ± 0.4	1.4 ± 0.4
Ventricular rate (/min) [mean, ± SD]	71.9 ± 12.5	73.1 ± 13.7
Atrial rate (/min) [mean, ± SD]	72.2 ± 15.1	74.6 ± 23.1
Current smoker (%)	3417 (35.6%)	185 (42.9%)
Current drinker (%)	2214 (23.0%)	105 (24.4%)

Student’s t-test or Mann–Whitney U test for continuous variables, Chi-squared or Fisher’s exact test for categorical variables

*Abbreviations*: *BMI* Body Mass Index, *SBP* Systolic Blood Pressure, *DBP* Diastolic Blood Pressure, *GLU* glucose, *eGFR* Estimated Glomerular Filtration Rate, *CR* creatinine, *WBC* White Blood Cell, *UA* Uric Acid, *BUN* Blood Urea Nitrogen, *AST* Aspartate aminotransferase, *LDL* low-density lipoprotein, *HDL* high-density lipoprotein

### Performance of traditional ASCVD prediction models

**Fig. 2:**
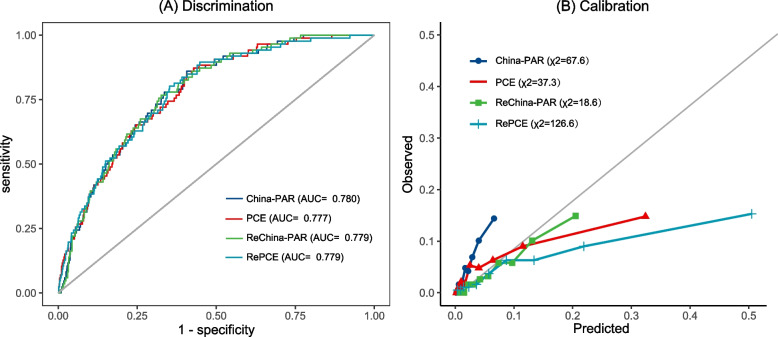
Discrimination and Calibration of Contemporary Prediction Models in Our Cohort. Discrimination and calibration of contemporary prediction models in each cohort. **A** Receiver operating characteristic curve (AUC) analysis for contemporary prediction models. **B** Hosmer–Lemeshow calibration plots of contemporary prediction models. Abbreviations: ReChina-PAR, Recalibrated China-PAR; RePCE, Recalibrated PCE

Figure 2 depicted the discrimination and calibration of the PCE (White) and China-PAR models before and after recalibration. All models showed moderate discrimination, and the highest discrimination was showed in the China-PAR model with an AUC of 0.780. However, all models exhibited poor calibration with a Hosmer–Lemeshow χ2 value greater than 18 (*p* < 0.05). The Brier score was between 0.043 and 0.057 and MCC was between 0.186 and 0.194 (Table 2).

**Table 2:** Performance of risk prediction models in the test cohort

**Algorithms**	**Overall**		**Discrimination**	**Calibration**	**Clinical Usefulness**
	**Brier**	**MCC**	**AUC (95%CI)**	**Hosmer–Lemeshow χ2 (** ***p*** ** value)**	**Net benefit at threshold of 5%**
PCE (White)	0.045	0.186	0.777 (0.733–0.821)	37.3 (*p* < 0.01)	0.013
China-PAR	0.043	0.191	0.780 (0.737–0.822)	67.6 (*p* < 0.001)	0.016
RePCE (White)	0.057	0.194	0.779 (0.734–0.825)	126.6 (*p* < 0.001)	0.016
ReChina-PAR	0.043	0.193	0.780 (0.737–0.822)	18.6 (*p* < 0.05)	0.017
ANN	0.041	0.218	0.800 (0.759–0.838)	9.1 (*p* = 0.33)	0.017
RF	0.042	0.181	0.759 (0.713–0.804)	12.6 (*p* = 0.13)	0.011
GBM	0.042	0.193	0.774 (0.727–0.820)	12.1 (*p *= 0.15)	0.013
KNN	0.042	0.175	0.767 (0.723–0.811)	34.7 (*p *< 0.01)	0.015
Adaboost	0.051	0.163	0.727 (0.679–0.775)	136.4 (*p *< 0.001)	0.010
SVM	0.043	0.145	0.697 (0.642–0.752)	4.0 (*p* = 0.86)	0.009
Catboost	0.041	0.206	0.787 (0.745–0.830)	10.2 (*p* = 0.25)	0.015

*Abbreviations*: *ASCVD* atherosclerotic cardiovascular disease, *CI* confidence interval, *MCC* Matthews correlation coefficient, *AUC* area under the receiver operating characteristic curve, *PCE* Pooled Cohort Equations, *China-PAR* Prediction for ASCVD Risk in China, *RePCE* Recalibrated PCE, *ReChina-PAR* Recalibrated China-PAR, *ANN* Artificial Neural Network, *RF* Random Forest, *GBM* Gradient Boosting Machine, *KNN* K Nearest Neighbor, *Adaboost* Adaptive Boosting, *SVM* Support Vector Machine, *Catboost* Categorical Boosting

### Compared of ML-based ASCVD and traditional models

**Fig. 3:**
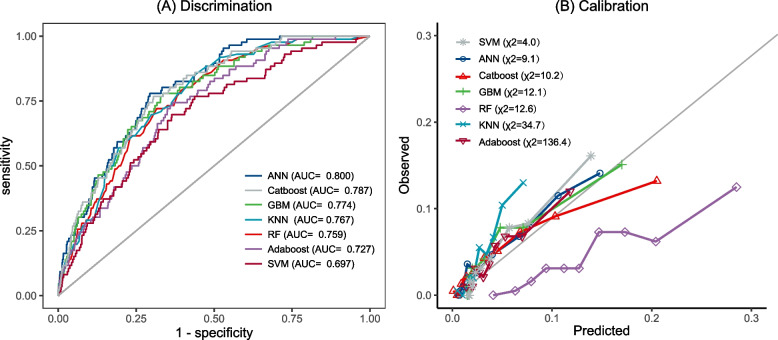
Discrimination and Calibration of Machine Learning-based ASCVD Models in Test Cohort. Discrimination and calibration of machine learning-based ASCVD prediction models in test cohort. **A** Receiver operating characteristic curve (AUC) analysis for machine learning-based ASCVD prediction models. **B** Hosmer–Lemeshow calibration plots of machine learning-based ASCVD prediction models. Abbreviations: ASCVD, Atherosclerotic cardiovascular disease; ANN, Artificial Neural Network; RF, Random Forest; GBM, Gradient Boosting Machine; KNN, K Nearest Neighbor; Adaboost, Adaptive Boosting; SVM, Support Vector Machine; Catboost, Categorical Boosting

**Fig. 4:**
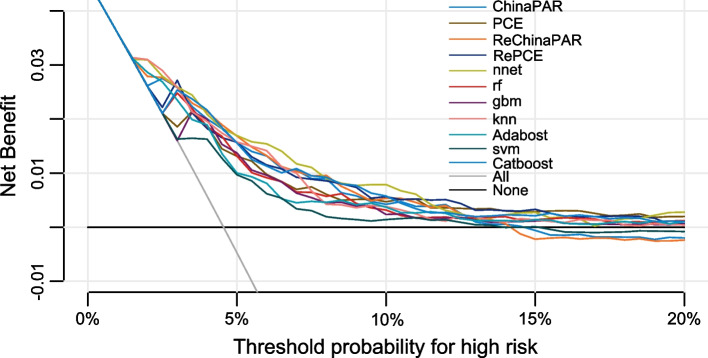
Decision Curves for PCE, China-PAR and Machine Learning-based Models. Abbreviations: PCE, Pooled Cohort Equations; ReChina-PAR, Recalibrated China-PAR; ANN, Artificial Neural Network; RF, Random Forest; GBM, Gradient Boosting Machine; KNN, K Nearest Neighbor; Adaboost, Adaptive Boosting; SVM, Support Vector Machine; Catboost, Categorical Boosting

Through stepwise model building and RFE algorithm, the final ML-based ASCVD models were reduced to 30 key predictor variables (Table 3). Figure 3 depicted the comparison of discrimination and calibration between the established ML classifiers. As shown, the ANN algorithm outperformed other classifiers and had the greatest AUC value and consistency. The AUC value of the ANN model was 0.800, which was higher than that in the China-PAR and PCE model (*p* = 0.12, 0.08). Calibration of the ANN model showed a significant improvement, the Hosmer–Lemeshow χ2 value was 9.1 (*p* = 0.33), and the Brier score and MCC of the ANN model were respectively 0.041 and 0.216, indicating a superior overall performance of the ANN model than traditional regression models. Decision Curve Analysis (DCA) demonstrated that ANN model provided a greater net benefit within a range of thresholds (Fig. 4). When the threshold was 5%, the ANN model had the greatest net benefit value of 0.017 among all models (Table 2). We also assess the NRI when using ML models compared to the traditional models, ANN model correctly classified more events and more non-events than China-PAR, PCE, Recalibrated China-PAR, and Recalibrated PCE (NRI: 0.355, 0.089, 0.088, 0.098, all *p* < 0.05) (Table 4).

**Table 3:** Predictor variables in ASCVD models

**Rank**	**ANN-based ASCVD prediction model**	**One minus AUC after permutations**
1	Age	0.6458121
2	SBP	0.7290980
3	V2.R Area	0.7464859
4	V2.Max R Amplitude	0.7522701
5	I.T Area (Full)	0.7565553
6	V2.S Area	0.7570991
7	V4.Max S Amplitude	0.7575463
8	V3.QRS Area	0.7575922
9	CR	0.7578144
10	I.T Duration	0.7582132
11	V6.T Area (Full)	0.7585335
12	V6.T Area	0.7590182
13	eGFR	0.7591497
14	I.T Peak Amplitude	0.7594988
15	GLU	0.7600205
16	V2.Max S Amplitude	0.7602828
17	V3.Max S Amplitude	0.7606117
18	V2.QRS Area	0.7607344
19	V6.Max R Amplitude	0.7615041
20	Peak E Wave Velocity	0.7618305
21	WBC	0.7618512
22	UA	0.7618903
23	I.P Area (Full)	0.7619013
24	aVR.T Area	0.7619031
25	DBP	0.7619409
26	V1.QRS Area	0.7619643
27	V3.S Area	0.7620318
28	I.T Area	0.7622241
29	V2.T Duration	0.7623373
30	V6.QRS Area	0.7628234

The importance of each feature was quantified using the permutation feature importance method which measures the importance of a feature by calculating the decrease in the model’s performance (area under the ROC curve) after permuting its values. The higher their values, the more important the feature is. Features are sorted according to permutation importance

*Abbreviations*: *ANN* Artificial Neural Network, *SBP* Systolic Blood Pressure, *CR* creatinine, *eGFR* Estimated Glomerular Filtration Rate, *GLU* glucose, *WBC* White Blood Cell, *UA* Uric Acid, *DBP* Diastolic Blood Pressure

**Table 4:** Net reclassification improvement (NRI) in the test set

**Ref**	**PCE**	**China-PAR**	**RePCE**	**ReChina-PAR**
**ANN**	0.089 * **(0.0104–0.1667)**	0.355 *** **(0.249–0.462)**	0.098 ** **(0.033–0.162)**	0.088 * **(0.017–0.158)**
RF	0.005 (-0.094–0.104)	0.332 *** (0.225–0.440)	0.036 * (-0.07–0.138)	0.005 (-0.095 0.106)
GBM	0.003 (-0.083–0.089)	0.299 *** (0.195–0.404)	0.093 * (0.010–0.176)	0.042 (-0.048–0.133)
KNN	-0.150 ** (-0.259–0.041)	0.085 * (0.008–0.162)	0.034 * (-0.067–0.134)	-0.003 (-0.105–0.098)
Adaboost	-0.312 *** (-0.414–0.211)	0.160 *** (0.103–0.217)	-0.333 *** (-0.395–0.271)	-0.327 *** (-0.396–0.258)
SVM	-0.110 (-0.232–0.0120)	0.189 *** (0.086–0.293)	-0.087 (-0.180–0.006)	-0.066 (-0.172–0.041)
Catboost	0.017 (-0.069–0.105)	0.264 *** (0.159–0.369)	0.072 (-0.003–0.147)	0.072 (-0.012–0.157)

*Abbreviations*: *PCE* Pooled Cohort Equations, *China-PAR* Prediction for ASCVD Risk in China, *RePCE* Recalibrated PCE, *ReChina-PAR* Recalibrated China-PAR, *ANN* Artificial Neural Network, *RF* Random Forest, *GBM* Gradient Boosting Machine, *KNN* K Nearest Neighbor, *Adaboost* Adaptive Boosting, *SVM* Support Vector Machine, *Catboost* Categorical Boosting; *P < 0.05; **P < 0.01; ***P < 0.001

### Variable importance

**Fig. 5:**
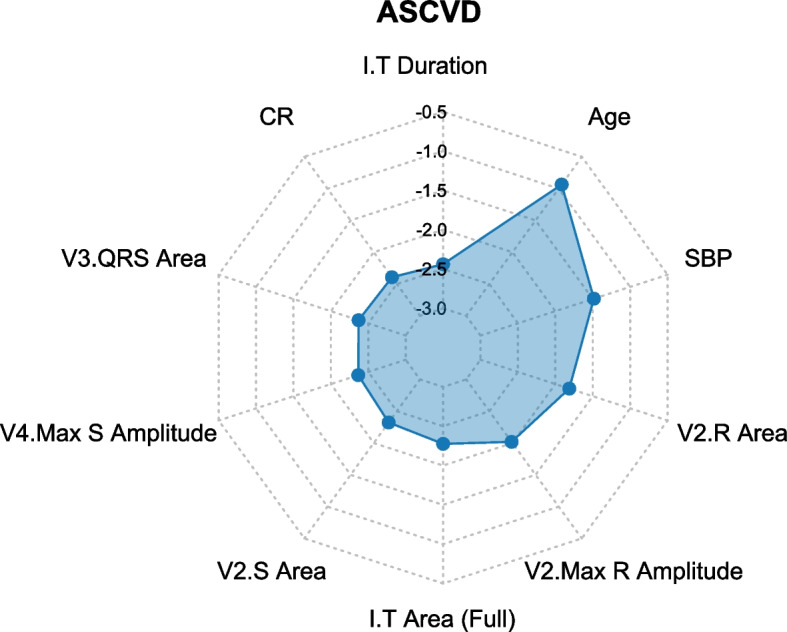
Radar Plot for the Ten Most Important Predictors of ASCVD. As the values of feature importances were spread over a wide range (more orders of magnitude), base-10 logarithmic transformation was performed to facilitate plotting

The leading predictors of the ANN ASCVD model are shown in Fig. 5 and a complete table of feature importance is available in Table 3. Age, SBP, R Area in V2, Max R Amplitude, and I.T Area (Full) in V2 were the most significant features to predict ASCVD.

## Discussion

In a community-based general population which included 11,956 adults from Northeast China, we developed seven ML models based on different algorithms. After extensive evaluation, the ANN model was chosen as the best model. The ANN model includes 30 predictors which can accurately and efficiently predict 5-year ASCVD in individuals with no history of CVD. Compared to the traditional regression models (China-PAR and PCE), the ANN model showed higher discrimination, better calibration, net benefit, and improved NRI in predicting ASCVD. Besides, our study provided a ranking of candidate variables by their significance in predicting ASCVD, which may help ASCVD risk stratification and management.

Early detection of high-risk individuals is the most effective approach to reduce the escalating incidence of ASCVD across multiple countries, while significant improvements of available prediction models were absent [34]. The PCE integrates several cardiovascular risk predictors to assess an individual’s 10-year risk of ASCVD and to guide treatment decisions. Disparities on distribution of cardiovascular risk factors existed between Asian and Western populations [35], clinical decisions may be influenced by over- or underestimation of risk. PCE provided moderate discrimination in the Korean KHS cohort, absolute 10-year ASCVD risk was overestimated by 56.5% for men and underestimated by 27.9% for women [8]. In the CHERRY Study in Southeastern China, PCE overestimated the risk by 63% in men and underestimated the risk by 34% in women [9]. In the “stroke belt” of Northern China, Fangshan Cohort Study found PCE showed an underestimate of 76.2% for men and 88.2% for women with poor calibration [10].

The China-PAR model was derived from multiple contemporary Chinese cohorts, and external validation studies of the model are limited. In the CHERRY study, the China-PAR model underestimated by 20% in men and 40% in women [9]. In the Fangshan Cohort, the China-PAR model overestimated by 29.4% risk in women [10]. When PCE and China-PAR models were applied in our cohort, we found that PCE overestimated by 63.8% in men and inversely underestimated the risk by 10.3% in women. Meanwhile, the China-PAR model underestimated the risk by 55.5% in men and 52.4% in women. After recalibration, the recalibrated PCE overestimated the risk by 144.8% in men and 129.9% in women, and the recalibrated China-PAR model inversely overestimated the risk by 98.3% in men and 64.7% in women. All PCE and China-PAR models had poor calibration, despite good discrimination. A potential reason for the differences in diverse Chinese populations is the regional disparity [36]. Residents in Northeast China tend to have a diet with high sodium and fat, which leads to the high prevalence of ASCVD up to 12.6% in this area [37].

These contemporary ASCVD risk calculators are parsimonious models based on a limited number of clinical risk variables, the potential influence of intricate and hidden interactions between weaker predictors may be overlooked. With the extension of artificial intelligence, ML algorithms have emerged as highly effective methods for resolving medical prediction puzzles in large-scale datasets and to allow guideline-directed management based on risk assessment [38]. Same as previous studies [25, 39–41], when compared with conventional models, our ANN-based ASCVD prediction model exhibited improved prediction performance (Table S4). The AUC of the ANN model was + 0.023, + 0.02, + 0.021, + 0.02 compared to that of PCE (White), China-PAR, Recalibrated PCE, and Recalibrated China-PAR (*p* = 0.08, 0.12, 0.12, 0.12), while calibration was significantly better (HL χ2 = 9.1 vs. 37.3, 67.6, 126.6, 18.6). In addition, DCA that accounts for the influence of false-negative (undertriage) and false-positive (overtriage), suggests an increased net clinical benefit with use of the ANN model as compared to traditional models if the ideal risk threshold for medical consultation lies between 5 and 10%. NRI also highlight the ability of ANN model to augment ASCVD prediction and provide a better risk stratification strategy for patients.

The observed incremental gains compared to traditional methods demonstrate the potential value of machine learning algorithms. The major advantage of ML algorithms over linear models is their capacity to capture the complex underlying interactions of myriad features and improve ex-sample predictions [42]. Althought the ML algorithms are inherently complex and difficult to interpret, ML models remain attractive due to their more accurate predictive power and the capacity to assimilate and evaluate large amounts of complex healthcare data.

### Limitations

Our study has several limitations. First, the ML algorithm cannot assess the independent effects of each variable on events and may be difficult to identify specific treatments to reduce individual risk. However, as the volume of data increases, ML algorithms allow for more in-depth prospective studies to identify the causative factors and interaction mechanisms. Second, longer follow-up is needed considering the chronic, progressive course of ASCVD. Third, we excluded 84 variables with > 10% missing data that might have predictive value, and the imputation of missing data might bias the analysis. However, the imputation by Multiple Imputation (MI) is known to be a precise method for imputation. Fourth, external validation studies are required to demonstrate the accuracy of the model’s predictions in diverse populations. Ultimately, the models were established using the initial follow-up blood pressure and glucose, but it may have changed during the follow-up period and this was not taken into account in the model.

## Conclusion

In this contemporary cohort of Northeast China, we observed that the PCE and China-PAR models provided adequate discrimination but poor calibration in predicting 5-year ASCVD risk. However, the ANN-based model incorporating 30 clinical variables outperformed PCE and China-PAR, even after recalibration. Our study highlights the limitations of traditional risk prediction models for ASCVD and demonstrates the potential of machine learning algorithms in improving risk prediction accuracy. Further studies are required to demonstrate the benefits of ML algorithms and to enhance the clinician's triage decision making.

## Supplementary Information

**Additional file 1. **Health Survey Form for Rural Residents in Liaoning Province (Version 2012).

**Additional file 2: Table S1.** List of Candidate Variables Used in this Study. **Table S2.** Results of Hyper-parameter Tuning with Different Algorithms in ML-based ASCVD – Models. **Table S3.** Estimation of Five-year ASCVD Risk Using the PCE (White) and China-PAR Models. **Table S4.** Comparison with Similar Studies. **Figure S1.** Methodology Flow Chart of Data Analysis.

## Data Availability

The datasets used and/or analysed during the current study are available from the corresponding author on reasonable request.

## Abbreviations

ASCVD: Atherosclerotic cardiovascular disease
CHD: Coronary Heart Disease
CVD: Cardiovascular Disease
PCE: Pooled Cohort Equations
China-PAR: Prediction for ASCVD Risk in China
TC: Total Cholesterol
HDL: High-density Lipoprotein
ECG: Electrocardiography
Echo: Echocardiography
LVEF: Left Ventricular Ejection Fraction
ML: Machine learning
WHO-QOL-BREF: WHO Quality of Life-BREF
PHQ-9: Patient Health Questionnaire-9
NCRCHS: Northeast China Rural Cardiovascular Health Study
BMI: Body Mass Index
BP: Blood pressure
MI: Myocardial Infarction
RFE: Recursive Feature Elimination
AUC: Mean Area under the Receiver Operating Characteristic Curve
ANN: Artificial Neural Network
RF: Random Forest
GBM: Gradient Boosting Machine
KNN: K Nearest Neighbors
AdaBoost: Adaptive Boosting
SVM: Support Vector Machine
CatBoost: Categorical Boosting
MCC: Matthews Correlation Coefficient
DCA: Decision-curve Analysis
