# Supplementary material for: Comparing the performance of machine learning and conventional models for predicting atherosclerotic cardiovascular disease in a general Chinese population
Source: BMC Med Inform Decis Mak. 2023 Jul 24;23:134. doi: 10.1186/s12911-023-02242-z (PMC10367272; doi:10.1186/s12911-023-02242-z)
Supplement: Supplementary file 1 — Additional file 1. Health Survey Form for Rural Residents in Liaoning Province (Version 2012). [file 12911_2023_2242_MOESM1_ESM.docx]

This questionnaire contains confidential information and is intended solely for internal use by the research team. The questionnaire consists of 13 pages. Please complete it carefully. Thank you for your support and cooperation!

**Health Survey Form for Rural Residents in Liaoning Province (Version 2012)**

Resident ID：□□□ □□□□ Start time of survey:___ h ___ min

**A. General Information**

A1 Name___________ A2 Contact phone number______________

A3 _____ County_____ Township _____ Village_____ Community _____ Building

A4 Gender Male=1 Female=2

Are you a woman who has experienced menopause?

Yes=1 No=2

If yes, what age did you experience menopause_____

A5 Date of Birth ___ Year ___Month ___Day

A6 What is your current marital status?

Never married=1 Married=2 Remarried=3 Divorced=4 Widowed=5

A7 What is your educational level?

Illiterate=1 Elementary school=2 Junior high school=3 Senior high school or technical school=4 Junior college=5 University or above=6

A8 What is your ethnicity?

Han=1 Mongolian=2 Korean=3 Hui=4 Manchu=5 Other=6

A9 Number of permanent residents in the household

___ people, Number of children ___ , Number of labor force___

A10 How long have you lived in the local area (survey location)?

Less than 5 years=1 Between 5 and 10 years=2 More than 10 years=3

**B. Economic status**B1 What was your household's net income in the past year, including government subsidies?

___ yuan/year

B2 What do you think is the per capita income level of your household compared to others in the local area? High=1 Medium=2 Low=3 Poverty=4

B3 What was the total amount spent on medical expenses and healthcare products for your entire household (including permanent residents) in the past year?

___ yuan/year

B4 Has your household been identified as a poverty-stricken or subsistence allowance household by the local government?

Yes=1 No=2

**C. Medical History**

| The form does not allow blank fields (multiple diseases can be selected). | | C1.1-1.9 Cardiovascular diseases | C2.1-2.8 Stroke | C3.1-3.9 Kidney Disease | C4.1-4.5 Diabetes |
| --- | --- | --- | --- | --- | --- |
| Do you have this disease:  (Yes=1, No=2) | |  |  |  |  |
| Disease specific subtypes (see notes) | 1 |  |  |  |  |
|  | 2 |  |  |  |  |
|  | 3 |  |  |  | --- |
|  | 4 |  |  |  | --- |
|  | 5 |  |  |  | --- |
|  | 6 |  | --- |  | --- |
| Date of diagnosis: (year-month) | |  |  |  |  |
| Diagnosis Hospital: (see notes) | |  |  |  |  |

**Notes:** **Cardiovascular Diseases** are classified as follows:

angina pectoris = 1 myocardial infarction = 2 atrial fibrillation = 3 other types of arrhythmias = 4 heart failure = 5 other = 6

**Stroke** is classified as follows:

cerebral hemorrhage = 1 cerebral infarction (including cerebral thrombosis and cerebral embolism) = 2 subarachnoid hemorrhage = 3 transient ischemic attack (TIA) = 4 unknown = 5

**Kidney Disease** is classified as follows:

nephritis (pyelonephritis, renal abscess, etc.) = 1 acute/chronic renal insufficiency = 2 kidney stones = 3 kidney tumors = 4 autoimmune kidney diseases (acute nephritis chronic nephritis nephrotic syndrome) = 5 other = 6

**Diabetes** is classified as follows:

type 1 = 1 type 2 = 2

**Diagnosing hospital:**

Provincial or above = 1 City-level = 2 County-level = 3 Township-level = 4 Village clinic = 5

C5 Have you ever had a coronary angiography?

Yes = 1 No = 2

C6 Have you ever had a coronary PCI procedure?

Yes = 1 No = 2

C7 Have you ever had a coronary artery bypass grafting (CABG) procedure?

Yes = 1 No = 2

C8 Have you experienced the following symptoms?

Yes = 1, No = 2: Exertional dyspnea__ Nocturnal dyspnea__ Reduced urine output/leg edema__ Decreased exercise tolerance__ Inability to sleep lying flat__

C9 Apart from the diseases listed above, have you ever had any other illnesses:______

Yes = 1 (please fill in the blank with the name of the illness) No = 2

**D. Family History**

|  | D1.1-1.10  Father | | D1.1-1.10  Mother | | D3.1-3.15  Siblings | | | D4.1-4.15  Children | | |
| --- | --- | --- | --- | --- | --- | --- | --- | --- | --- | --- |
| Family Medical History of Diseases  (Yes=1, No=2) | Medical conditions | Age of first onset | Medical conditions | Age of first onset | Medical conditions | Number | Age of first onset | Medical conditions | Number | Age of first onset |
| Diabetes |  |  |  |  |  |  |  |  |  |  |
| Hypertension |  |  |  |  |  |  |  |  |  |  |
| Coronary Heart Disease |  |  |  |  |  |  |  |  |  |  |
| Stroke |  |  |  |  |  |  |  |  |  |  |
| Atrial Fibrillation |  |  |  |  |  |  |  |  |  |  |

**E. High blood pressure-related information**

E1 What do you think is the normal blood pressure range for healthy adults?

1. Know ___ /___ mmHg 2. Do not know

E2 How long ago did a healthcare professional measure your blood pressure?

<6 months ago=1 6 months to 1 year ago=2 1 to 2 years ago=3 More than 2 years ago=4 Never measured=5 Cannot remember=6

E3 Have you been informed by a healthcare professional that you have hypertension (high blood pressure)? Yes=1 No=2 (If "No", please proceed directly to Part F)

E4 Initial diagnosis healthcare institution:

City, prefecture-level or higher hospital=1 County hospital=2 Township health center=3 Village clinic, private doctor=4

E5 Date of initial diagnosis ___ Year ___ Month, Blood pressure measurement: Systolic pressure ___mmHg, Diastolic pressure ___ mmHg

E6 Did you have a follow-up visit after your initial diagnosis?

Yes=1 No=2 (If "Yes", please proceed to E8)

E7 Reasons for not having a follow-up visit after initial diagnosis: (Multiple choices allowed)

Yes=1 No=2

E7.1 Do not consider hypertension to be a serious condition __

E7.2 Financial difficulties __

E7.3 Busy with work and no time __

E7.4 Inconvenient transportation and afraid of trouble __

E7.5 Other reasons __

E8 Number of visits including the initial diagnosis __ times

E9 Do you think hypertension poses a serious threat to your life?

Does not matter=1 Somewhat important=2 Very important=3

E10 Do you regularly measure your blood pressure after being diagnosed with hypertension?

At least once a week=1 At least once a month=2 At least once every three months=3 Once every six months=4 Once every six months or longer=5 Have not measured it again=6

E11 Have you been taking antihypertensive medication in the past two weeks?

Yes=1 No=2

E12 Can you take antihypertensive medication under the guidance of a doctor? (If not taking medication, please proceed to E17)

Regularly (more than 9 months/year) = 1 Intermittently (3 months-9 months/year) = 2

Occasionally (less than 3 months/year) = 3 Not taking medication = 4

E13 Effect of taking antihypertensive medication

**Good** (Blood pressure recorded below 140/90mmHg for more than 3/4 of the year) = 1

**Fair** (Blood pressure recorded below 140/90mmHg for more than 1/2 but less than 3/4 of the year) = 2 **Poor** (Blood pressure recorded above 140/90mmHg for more than 1/2 of the year) = 3 **Not measured** = 4

E14 Medication adherence

Have you ever forgotten to take your medication? Yes=1 No=2 Not sure=3

Have you ever been careless about taking your medication? Yes=1 No=2 Not sure=3

Have you ever stopped taking medication when you felt better? Yes=1 No=2 Not sure=3

Have you ever stopped taking medication when you felt worse? Yes=1 No=2 Not sure=3

Have you refused to take medication because of concerns about side effects? Yes=1 E No=2 Not sure=3

E15 Name of antihypertensive medication and average annual dosage

| Antihypertensive medications | Diuretics | ACEI | CCB | β-Blockers | Combination antihypertensive drugs | Others |
| --- | --- | --- | --- | --- | --- | --- |
| Medication name |  |  |  |  |  |  |
| Average amount (bottles/year) |  |  |  |  |  |  |
| Dosage (tablets/time) |  |  |  |  |  |  |
| Frequency (times/day) |  |  |  |  |  |  |

E16 Average annual cost of antihypertensive medication in RMB ___ (please proceed to section F)

E17 Reasons for not taking antihypertensive medication: (multiple choices allowed)

| Do E17.1-E17.5 exist | Insufficient understanding of the harms caused by hypertension. | Economic difficulties. | Lack of guidance on medication from a doctor. | Inconvenience or frequently forgetting to take the medication. | Others |
| --- | --- | --- | --- | --- | --- |
| Yes=1, No=2 |  |  |  |  |  |

**F. Awareness of blood lipids and blood sugar**

F1 When was the last time you had your blood sugar measured in a hospital? (If you have never had it measured, please go to F4).

Never had it measured = 1 <6 months ago = 2 6 months to 1 year ago = 3 1 to 2 years ago = 4 2 years or more ago = 5 can't remember = 7

F2 Has a doctor told you that you have diabetes?

Yes = 1 (if "Yes", please answer F3) No = 2

No, but it is considered pre-diabetes / borderline diabetes = 3

F3 If you are aware that you have diabetes, have you been taking medication to control your condition in the past two weeks?

Yes = 1 No = 2

F4 When was the last time you had your blood lipids measured in a hospital? (If you have never had it measured, please go to section G)

Never had it measured = 1 <6 months ago = 2 6 months to 1 year ago = 3 1 to 2 years ago = 4 2 years or more ago = 5 can't remember = 7

F5 Has a doctor told you that your blood lipids are elevated?

Yes = 1 (if "Yes", please answer F6) No = 2 Do not know = 3

F6 If you are aware that your blood lipids are elevated, have you been controlling them through diet, exercise, or medication in the past two weeks?

Yes = 1 No = 2

**G. Smoking Status**

G1 Have you ever smoked? (i.e., smoked at least one cigarette per day, continuously or cumulatively, for 6 months or more)

Yes = 1 No = 2

(If "Yes", please continue to answer the following questions. If "No", please proceed to Section H.)

G2 Do you currently smoke?

Yes = 1 No = 2

G3 At what age did you start smoking?

__years

G4 On average, how many cigarettes do you smoke per day or how much dry tobacco do you smoke per week?

_______

G5 Have you ever quit smoking?

Yes=1 No=2 (If "No", please proceed to section H)

G6 For those who have quit smoking or are currently quitting, what is the cumulative time you have quit smoking?

<6 months=1 6 months or more=2 1 year or more=3 2 years or more=4 Can't remember=5

**H. Alcohol Consumption**

H1 Do you drink regularly? (i.e., at least twice a week for more than one year)

Yes=1 No=2 (If "Yes", please answer the following questions. If "No", please proceed to Part I)

H2 Do you still drink alcohol now?

Yes=1 No=2

H3 At what age did you start drinking regularly?

___ years

H4 Excluding the years you did not drink, how many years have you been drinking regularly? ___ years

H5 What types of alcoholic beverages do you drink, how often, and how much per occasion?

| Category | Beerr | Baijiu | wine |
| --- | --- | --- | --- |
| Frequency | ( ) Per week | ( ) Per week | ( ) Per week |
| Alcohol intake. | ( ) Bottle/time | ( ) Bottle/time | ( ) Bottle/time |

H6 Have you ever quit drinking?

Yes=1 No=2 (If "No", please proceed to section I)

H7 If you have quit drinking or are currently quitting, how long have you been sober?

<6 months=1 6 months or more=2 1 year or more=3 2 years or more=4 Can't remember=5

**I. Lifestyle (in the past year)**

I1 How many fresh vegetables do you eat per week on average?

Rarely eat = 1 Less than 2 catties = 2 2-3 catties = 3 3-4 catties = 4 More than 4 catties = 5

I2 How much lean meat, fish, and poultry do you eat on average per week?

rarely eat=1, less than 0.5 kg=2, 0.5-1 kg=3, more than 1 kg=4

I3 How much legumes or soy products do you eat on average per week?

Rarely=1 2-3 times=2 4 times or more=3

I4 How often do you eat greasy foods (fatty meat, fried foods) on average per week?

Rarely=1 2-3 times=2 4 times or more=3

I5 How many times do you drink tea per day on average (if "1", please skip to I6)?

Do not drink = 1 Occasionally drink = 2 1-2 times per day = 3 3 times or more per day = 4

Do you like to drink strong tea?

Yes = 1 No = 2

I6 On average, how many times a day do you eat pickles or soybean paste?

Rarely eat=1, 2-3 times=2, 3 times or more=3.

I7 What is your average monthly consumption of salt in your household for food?

___ catty/month

How much salt do you use for pickling salted vegetables or making soybean paste in a year?

___ catty/year

I8 How much lard does your family purchase or refine per month?

___ jin/month

I9 What is your average nightly sleep time and nap time? Please provide your average nightly sleep time and nap time in hours (total time divided by 7 days).

___ hours ___ hours

I10 What is the intensity level of physical activity in your work or occupation?

1= Light (sitting or standing for 75% of the time, and standing activity for 25% of the time, such as office work, repairing electrical appliances and clocks, salespersons, hotel servers, chemical experiment operations, teaching, etc.)

2= Moderate (sitting or standing for 25% of the time, and special occupational activities for 75% operation, metal cutting, etc.)

3= Heavy (sitting or standing for 40% of the time, and special occupational activities for 60% of the time, such as non-mechanized agricultural labor, loading and unloading, steelmaking, dance, sports, mining, etc.)

I11 Do you exercise regularly?

Yes=1 No=2 (If "No", please proceed to I12)

If yes, how often and how long do you exercise per week on average?

____ times/week ____ minutes each time

What is your most common exercise method? (Single choice)

Walking=1 Running=2 Swimming=3 Ball sports=4 Qigong=5 Mountaineering=6 Other=7

I12 Have you taken any medication in the past 2 weeks?

Yes=1 No=2 (If "No", please proceed to section J)

If yes, what medication have you taken: __________

**J. OSAS Screening**

J1 Do you snore when you sleep?

Yes=1 No=2 Not sure=3 (If "No" or "Not sure", please proceed to section K)

J2 How loud is your snoring usually?

Slightly louder than breathing=1 As loud as talking=2 Louder than talking=3

Very loud, can be heard in the next room=4

J3. Do you experience breathing pauses while snoring during sleep?

Yes=1 No=2 Not sure=3

J4 Have you ever woken up feeling breathless during sleep?

Yes=1 (If "Yes", please answer J5) No=2 Not sure=3

J5 If yes, how often does it happen?

Almost every day=1 3-4 times a week=2 1-2 times a week=3 1-2 times a month=4

Never or almost never=5

J6 Do you feel excessively sleepy during the day?

Yes=1 No=2

**K. Depression Screening (PHQ-9)**

Instructions:

For each of the following 9 questions, please circle the number that best applies to how often you have been bothered by the particular problem over the last 2 weeks.

K1 Little interest or pleasure in doing things

Not at all=0 Several days=1 More than half the days=2 Nearly every day=3

K2 Feeling down, depressed, or hopeless

Not at all=0 Several days=1 More than half the days=2 Nearly every day=3

K3 Trouble falling or staying asleep, or sleeping too much

Not at all=0 Several days=1 More than half the days=2 Nearly every day=3

K4 Feeling tired or having little energy

Not at all=0 Several days=1 More than half the days=2 Nearly every day=3

K5 Poor appetite or overeating

Not at all=0 Several days=1 More than half the days=2 Nearly every day=3

K6 Feeling bad about yourself — or that you are a failure or have let yourself or your family Not at all=0 Several days=1 More than half the days=2 Nearly every day=3

K7 Trouble concentrating on things, such as reading the newspaper or watching television

Not at all=0 Several days=1 More than half the days=2 Nearly every day=3

K8 Moving or speaking so slowly that other people could have noticed? Or the opposite — being so fidgety or restless that you have been moving around a lot more than usual

Not at all=0 Several days=1 More than half the days=2 Nearly every day=3

K9 Thoughts that you would be better off dead or of hurting yourself in some way

Not at all=0 Several days=1 More than half the days=2 Nearly every day=3

If you selected any of the questions in this questionnaire, how difficult have these problems made it for you to do your work, take care of things at home, or get along with other people?

No difficulty at all=1 Some difficulty=2 Quite a lot of difficulty=3 Extreme difficulty=4

**L. Quality of Life (WHOQOL-BREF)**

Instructions:

This assessment asks how you feel about your quality of life, health, and other areas of your life. Please answer all the questions. If you are unsure about which response to give to a question, please choose the one that appears most appropriate. This can often be your first response. Please read the question, assess your feelings, for the last four weeks, and circle the number on the scale for each question that gives the best answer for you.

|  |  | Very  poor | Poor | Neither poor nor good | Good | Very  good |
| --- | --- | --- | --- | --- | --- | --- |
| 1 | How would you rate your quality of life? | 1 | 2 | 3 | 4 | 5 |

|  |  | Very  dissatisfied | Fairly  Dissatisfied | Neither satisfied nor dissatisfied | Satisfied | Very satisfied |
| --- | --- | --- | --- | --- | --- | --- |
| 2 | How satisfied are you with your health? | 1 | 2 | 3 | 4 | 5 |
| The following questions ask about how much you have experienced certain things in the last four weeks. | | | | | | |
|  |  | Not  at all | A  Small amount | A  Moderate amount | A  great deal | An  Extreme amount |
| 3 | To what extent do you feel that physical pain prevents you from doing what you need to do? | 1 | 2 | 3 | 4 | 5 |
| 4 | How much do you need any medical treatment to function in your daily life? | 1 | 2 | 3 | 4 | 5 |
| 5 | How much do you enjoy life? | 1 | 2 | 3 | 4 | 5 |
| 6 | To what extent do you feel your life to be meaningful? | 1 | 2 | 3 | 4 | 5 |

|  |  | Not at all | Slightly | Moderately | Very | Extremely |
| --- | --- | --- | --- | --- | --- | --- |
| 7 | How well are you able to concentrate? | 1 | 2 | 3 | 4 | 5 |
| 8 | How safe do you feel in your daily life? | 1 | 2 | 3 | 4 | 5 |
| 9 | How healthy is your physical environment? | 1 | 2 | 3 | 4 | 5 |

|  |  | Not at all | Slightly | Somewhat | To a great extent | Completely |
| --- | --- | --- | --- | --- | --- | --- |
| 10 | Do you have enough energy for everyday life? | 1 | 2 | 3 | 4 | 5 |
| 11 | Are you able to accept your bodily appearance? | 1 | 2 | 3 | 4 | 5 |
| 12 | Have you enough money to meet your needs? | 1 | 2 | 3 | 4 | 5 |
| 13 | How available to you is the information you need in your daily life? | 1 | 2 | 3 | 4 | 5 |
| 14 | To what extent do you have the opportunity for leisure activities? | 1 | 2 | 3 | 4 | 5 |

|  |  | Not at all | Slightly | Moderately | Very | Extremely |
| --- | --- | --- | --- | --- | --- | --- |
| 15 | How well are you able to get around physically? | 1 | 2 | 3 | 4 | 5 |

The following questions ask you to say how good or satisfied you have felt about various aspects of your life over the over the last four weeks.

|  |  | Very Dissatisfied | Fairly  Dissatisfied | Neither  Satified nor Dissatisfied | Satisfied | Very satisfied |
| --- | --- | --- | --- | --- | --- | --- |
| 16 | How satisfied are you with your sleep? | 1 | 2 | 3 | 4 | 5 |
| 17 | How satisfied are you with your ability to perform your daily living activities? | 1 | 2 | 3 | 4 | 5 |
| 18 | How satisfied are you with your capacity for work | 1 | 2 | 3 | 4 | 5 |
| 19 | How satisfied are you with yourself? | 1 | 2 | 3 | 4 | 5 |
| 20 | How satisfied are you with your personal relationships? | 1 | 2 | 3 | 4 | 5 |
| 21 | How satisfied are you with your sex life? | 1 | 2 | 3 | 4 | 5 |
| 22 | How satisfied are you with the support you get from your friends? | 1 | 2 | 3 | 4 | 5 |
| 23 | How satisfied are you with the conditions of your living place? | 1 | 2 | 3 | 4 | 5 |
| 24 | How satisfied are you with your access to health services? | 1 | 2 | 3 | 4 | 5 |
| 25 | How satisfied are you with your transport? | 1 | 2 | 3 | 4 | 5 |

The following question refers to how often you have felt or experienced certain things in the last four weeks.

|  |  | Never | Infrequently | Sometimes | Frequently | Always |
| --- | --- | --- | --- | --- | --- | --- |
| 26 | How often do you have negative feelings such as blue mood, despair, anxiety or depression? | 1 | 2 | 3 | 4 | 5 |

Furthermore, there are three additional questions:

1．Does family conflict affect your life?

Not at all = 1 Very little = 2 Somewhat = 3 Quite a bit = 4 Extremely = 5

2．How is your appetite?

Very poor = 1 Poor = 2 Fair = 3 Good = 4 Very good = 5

3. If you were to rate your overall quality of life based on your physical health, mental health, social relationships, and environment, what score would you give yourself? (Scored from 0 to 100)

Score: __ (out of 100)

**The survey is now complete. Please check to make sure there are no omissions or errors!**

End of survey time:___ h ___ min

1. The respondent is: self=1 spouse=2 parent=3 child=4 other=5
2. The respondent's level of cooperation:

Very cooperative=1 Basically cooperative=2 Not very cooperative=3 Not cooperative at all=4

1. Signature of the interviewer: _________(required*)
2. Date of the survey: ___Year ___Month ___ Day (required*)
