# Supplementary material for: Comparing the performance of machine learning and conventional models for predicting atherosclerotic cardiovascular disease in a general Chinese population
Source: BMC Med Inform Decis Mak. 2023 Jul 24;23:134. doi: 10.1186/s12911-023-02242-z (PMC10367272; doi:10.1186/s12911-023-02242-z)

**Supplementary Material**

Comparing the performance of machine learning and conventional models for predicting atherosclerotic cardiovascular disease in a general Chinese population

Zihao Fan, Zhi Du, Jinrong Fu, Ying Zhou, Pengyu Zhang, Chuning Shi, Yingxian Sun*.

**Table S1. List of Candidate Features Used in this Study**

**Table S2. Results of Hyper-parameter Tuning with Different Algorithms in ML-based ASCVD Models**

**Table S3. Estimation of Five-year ASCVD Risk Using the PCE (White) and China-PAR Models**

**Table S4. Comparison with Similar Studies**

**Figure S1. Methodology Flow Chart of Data Analysis**

This supplementary material has been provided by the authors to give readers additional information about their work.

**Table S1. List of Candidate Variables Used in this Study**

| **Variable Categories** | **Variables** | |
| --- | --- | --- |
| **Demographics**, **behavioral and psychological variables**  **(n= 98)** | Sex | Mean Hipline |
|  | Age | Waist-to-Hip Ratio |
|  | Education | Mean SBP |
|  | DM Type1 | Mean DBP |
|  | DM Type2 | Mean Pulse |
|  | Family DM | WBC |
|  | Family HT | Hb |
|  | Family CHD | RBC |
|  | Family Stroke | HCT |
|  | Family AF | MCV |
|  | Ever Smoke | MCH |
|  | Current Smoke | MCHC |
|  | Ever Drink | PLT |
|  | Current Drink | MPV |
|  | Vegetable | PDW |
|  | Meat | PCT |
|  | Bean | ALT |
|  | Fat food | AST |
|  | Tea | AST/ALT |
|  | Pickles | BUN |
|  | Salt | CR |
|  | Food Salt | eGFR |
|  | Lard | UA |
|  | Night sleep | GLU |
|  | Noon sleep | TCH |
|  | Sleep Duration | TG |
|  | Physical Labor Intensity | HDL |
|  | Exercise | LDL |
|  | snoring | Ca |
|  | PHQ9 score 1-9 | P |
|  | PHQ9 additional question 10 | Mg |
|  | WHOQOL-BREF question 1-29 | K |
|  | Mean Height | Na |
|  | Mean Weight | Cl |
|  | BMI |  |
|  |  |  |
| **Variable Categories** | **Variables** | |
| **Electrocardiograph variables**  **(n= 444)** | ST at J Point in 12 Leads | T Area in 12 Leads |
|  | P Area in 12 Leads | T’ Area in 12 Leads |
|  | P′ Area in 12 Leads | T Area (Full) in 12 Leads |
|  | P Area (Full) in 12 Leads | T Peak Time in 12 Leads |
|  | P Peak Time in 12 Leads | T Peak Amplitude in 12 Leads |
|  | P′ Peak Time in 12 Leads | T’Peak Amplitude in 12 Leads |
|  | P Peak Amplitude in 12 Leads | T Duration in 12 Leads |
|  | P′ Peak Amplitude in 12 Leads | T’ Duration in 12 Leads |
|  | P Duration in 12 Leads | Minimum ST level in 12 Leads |
|  | P′ Duration in 12 Leads | Maximum ST level in 12 Leads |
|  | QRS Area in 12 Leads | Max S Amplitude in 12 Leads |
|  | Q Area in 12 Leads | P axis |
|  | Q Peak Amplitude in 12 Leads | QRS width |
|  | Q Duration in 12 Leads | QT interval |
|  | R Area in 12 Leads | PR interval |
|  | R’ Area in 12 Leads | QTC Count |
|  | R Peak Time in 12 Leads | R axis |
|  | R Duration in 12 Leads | T axis |
|  | R’ Duration in 12 Leads | Atrial rate |
|  | S Area in 12 Leads | Ventricular rate |
|  | S′ Area in 12 Leads | PR interval |
|  | S Peak Time in 12 Leads | QRS duration |
|  | S Duration in 12 Leads | Mean RR interval |
|  | S′Duration in 12 Leads |  |
| **Echocardiography variables**  **(n=9)** | Aortic diameter | |
|  | Interventricular septal thickness | |
|  | End-diastolic diameter of left ventricle | |
|  | Left ventricular end systolic diameter | |
|  | Left ventricular posterior wall thickness | |
|  | Left ventricular ejection fraction | |
| **Variable Categories** | **Variables** | |
|  | Mitral valve E peak velocity | |
|  | Mitral valve A peak velocity | |
|  | Aortic valve Flow velocity | |

Abbreviations: DM, Diabetes Mellitus; HT, Hypertension; CHD, Coronary Heart Disease; AF, Atrial Fibrillation; BMI, Body Mass Index; SBP, Systolic Blood Pressure; DBP, Diastolic Blood Pressure; WBC, White Blood Cell; Hb, Hemoglobin; RBC, Red Blood Cell; HCT, Red blood cell specific volume; MCV, Mean Corpuscular Volume; MCH, Mean Corpuscular Hemoglobin; MCHC, Mean Corpuscular Hemoglobin Concentration; PLT, Platelet count; MPV, Mean Platelet Volume; PDW, Platelet Distribution Width; PCT, Plateletcrit; ALT, Alanine aminotransferase; AST, Aspartate aminotransferase; BUN, Blood Urea Nitrogen; CR, creatinine; eGFR, Estimated Glomerular Filtration Rate; UA, Uric Acid; GLU, glucose; TCH, Total cholesterol; TG, triglyceride; HDL, high-density lipoprotein; LDL, low-density lipoprotein.

**Table S2. Results of Hyper-parameter Tuning with Different Algorithms in ML-based ASCVD Models**

| **Algorithms** | **Hyper-parameter space** | **Best Combination of Hyperparameters** | **AUC in the training cohort** | **AUC in the test cohort** |
| --- | --- | --- | --- | --- |
| Artificial Neural Network | {‘size’:[1,2,3,4,5,6,7,8,9,10,11,12,13,14,15,16,17,18,19,20],  ‘decay’:[0.1,0.2,0.3,0.4,0.5,0.6,0.7,0.8,0.9,1]} | {‘size’: 1,  ‘decay’: 1} | 0.793 | 0.800 |
| Random Forest | {‘mtry’:[1,2,3,4,5,6,7,8,9,10,20,30,40,50,60,70,80,90,100,200,300,400,500]} | {‘mtry’: 3} | 0.776 | 0.759 |
| Gradient Boosting Machine | {‘interaction.depth’:[1,2,3,4,5,6,7,8,9,10],  ‘n.trees’:[150,200,250,300,350],  ‘shrinkage’:[0.001,0.01,0.1],  ‘n.minobsinnode’:[5,10,20,30]} | {‘interaction.depth’: 9,  ‘n.trees’: 250’,  ‘shrinkage’: 0.01,  ‘n.minobsinnode’: 30} | 0.789 | 0.774 |
| K Nearest Neighbor | {‘k’:[445,446,447,448,449,450,451,452,452,454,455} | {‘k’: 448} | 0.777 | 0.767 |
| Adaptive Boosting | {‘nIter’:[100,110,120,130,140,150,160,170,180,190,200,210,220,230,240,250,260,270,280,290,300} | {‘nIter’: 200} | 0.760 | 0.727 |
| Support Vector Machine | {‘sigma’:[0.1,0.15,0.2,  C=[0.01,0.1,1} | {‘sigma’:0.1,  C=1} | 0.720 | 0.658 |
| Categorical Boosting | {‘depth’:[1,2,3,4,5,6,7,8,9,10],  ‘learning rate’:[ 0.05,0.1,0.3,0.5,1],  ‘iterations’:[100,200,300],  ‘l2_leaf_reg’:[ 0.0000001,0.000001,0.00001],  ‘rsm’:[ 0.8,0.9,1],  ‘border_count’:[250,255,260]} | {‘depth’:2,  ‘learning rate’:0.05,  ‘iterations’:100,  ‘l2_leaf_reg’,0.000001,  ‘rsm’:0.9,  ‘border_count’:255} | 0.788 | 0.787 |

Hyper-parameters not listed in the table were set to default values predefined in the caret package (version 6.0-90). Hyper-parameter tuning of each machine learning algorithm was performed with grid search. Models with each combination of hyper-parameters were trained with stratified 10-fold cross-validation on the training cohort, and the average area under the receiver operating characteristic curve (AUC) was calculated. Using the best performing combination of hyper-parameters, models were evaluated in the training cohort. Artificial Neural Network exhibited the best discriminative capability in training and test cohorts as well, therefore, it was used as the final model. Regarding the architecture of the ANN, the nnet function from R's caret package was used. The nnet function creates a feedforward neural network with three layers: an input layer, a hidden layer, and an output layer. Each neuron in the input layer is connected to each neuron in the hidden layer, and each neuron in the hidden layer is connected to each neuron in the output layer.

**Table S3. Estimation of Five-year ASCVD Risk Using the PCE (White) and China-PAR Models**

| **Model** | **S_0_(t) at 5 years** | **Mean Score** | **Equations for Individual Score** |
| --- | --- | --- | --- |
| **Men** |  |  |  |
| Original PCE (White) | 0.9625 | 60.11 | =12.344×ln(age)+11.853×ln(TC)-2.664×ln(age)×ln(TC)-7.990×ln(HDLC)+1.769×ln(age)×ln(HDLC) (+1.797×ln(SBP) if hypertension treated) (+1.764×ln(SBP) if hypertension untreated) (+7.837-1.795×ln(age) if current smoker) (+0.658 if diabetes) |
| Original China-PAR | 0.9724 | 142.11 | =31.97×ln(age)+0.62×ln(TC)-0.69×ln(HDLC)-0.71×ln(waist) (+[27.39-6.02×ln(age)]×ln(SBP) if hypertension treated) (+[26.15-5.73×ln(age)]×ln(SBP) if hypertension untreated) (+3.96-0.94×ln(age) if current smoker) (+6.22-1.53×ln(age) if having family history of ASCVD) (+0.36 if diabetes) (+0.48 if in Northern China) (-0.16 if living in urban) |
| Recalibrated PCE (White)  by NCRCHS | 0.9399 | 60.11 | =12.344×ln(age)+11.853×ln(TC)-2.664×ln(age)×ln(TC)-7.990×ln(HDLC)+1.769×ln(age)×ln(HDLC) (+1.797×ln(SBP) if hypertension treated) (+1.764×ln(SBP) if hypertension untreated) (+7.837-1.795×ln(age) if current smoker) (+0.658 if diabetes) |
| Recalibrated China-PAR  by NCRCHS | 0.9399 | 142.11 | =31.97×ln(age)+0.62×ln(TC)-0.69×ln(HDLC)-0.71×ln(waist) (+[27.39-6.02×ln(age)]×ln(SBP) if hypertension treated) (+[26.15-5.73×ln(age)]×ln(SBP) if hypertension untreated) (+3.96-0.94×ln(age) if current smoker) (+6.22-1.53×ln(age) if having family history of ASCVD) (+0.36 if diabetes) (+0.48 if in Northern China) (-0.16 if living in urban) |
| **Model** | **S_0_(t) at 5 years** | **Mean Score** | **Equations for Individual Score** |
| **Women** |  |  |  |
| Original PCE (White) | 0.9890 | -29.65 | =-29.799×ln(age)+4.884×ln(age)×ln(age)+13.540×ln(TC)-3.114×ln(age)×ln(TC)-13.578×ln(HDLC)+3.149×ln(age)×ln(HDLC) (+2.019×ln(SBP) if hypertension treated) (+1.957×ln(SBP) if hypertension untreated) (+7.574-1.665×ln(age) if current smoker) (+0.661 if diabetes) |
| Original China-PAR | 0.9841 | 119.03 | =24.87×ln(age)+0.06×ln(TC)-0.22×ln(HDLC)+1.48×ln(waist) (+[20.71-4.53×ln(age)] ×ln(SBP) if hypertension treated) (+[19.98 -4.36×ln(age)]×ln(SBP) if hypertension untreated) (+0.49 if current smoker) (+0.57 if diabetes) (+0.54 if in Northern China) |
| Recalibrated PCE (White)  by NCRCHS | 0.9609 | -29.65 | =-29.799×ln(age)+4.884×ln(age)×ln(age)+13.540×ln(TC)-3.114×ln(age)×ln(TC)-13.578×ln(HDLC)+3.149×ln(age)×ln(HDLC) (+2.019×ln(SBP) if hypertension treated) (+1.957×ln(SBP) if hypertension untreated) (+7.574-1.665×ln(age) if current smoker) (+0.661 if diabetes) |
| Recalibrated China-PAR  by NCRCHS | 0.9609 | 119.03 | =24.87×ln(age)+0.06×ln(TC)-0.22×ln(HDLC)+1.48×ln(waist) (+[20.71-4.53×ln(age)] ×ln(SBP) if hypertension treated) (+[19.98 -4.36×ln(age)]×ln(SBP) if hypertension untreated) (+0.49 if current smoker) (+0.57 if diabetes) (+0.54 if in Northern China) |

Equations for PCE (White) were obtained from the Supplementary eTable 1 in JAMA 2014; 311:1406–1415; Equations for China-PAR were obtained from the Supplemental Table 1 in Circulation 2016; 134:1430-1440. S**_0_** (t)=exp(- five-year Kaplan-Meier ASCVD rate). Final risk estimation was calculated as: Predicted ASCVD risk = 1 - S_0_(t)^e(Individual score – Mean score)^. Abbreviations: PCE, Pooled Cohort Equation; China-PAR, Prediction for ASCVD Risk in China; NCRCHS, Northeast China Rural Cardiovascular Health Study; TC, Total cholesterol; HDLC, high-density lipoprotein cholesterol.

**Table S4. Comparison with Similar Studies**

| **Author** | **Population** | **Techniques** | **Evaluation Metrics** | **Comparison Model** |
| --- | --- | --- | --- | --- |
| Ward, et. al 2020 (25) | multi-ethnic population | LRL2, LRLasso, RF, GBM, and XGBoost | AUC | PCE |
| Hathaway, et. al 2021 (40) | MSEA Study | COXPH, RSF, lSVM, nMTLR, and DeepSurv | AUC and NRI | PCE |
| Cho, et. al 2021 (39) | Korean adults | LR, RF, TB, AdaBoost, and ANN | AUC, Calibration Curve, and Net Benifit | PCE |
| Siva, et. al 2022 (41) | CLARIFY Study | LASSO-Cox and nomogram | AUC and PR Curve, | PCE |

Abbreviations: LRL2, Logistic Regression with the Standard L2 Penalty; LRLasso, Logistic Regression with the L1 Lasso Penalty; RF, Random Forest; GBM, Gradient Boosted Machines; XGBoost, Extreme Gradient Boosted Models; AUC, the Average Area under the Receiver Operating Characteristic Curve PCE, Pooled Cohort Equation; COXPH, Cox Proportional Hazards Model; RSF, Random Survival Forest; ISVM, Linear Support Vector Machines; nMTLR, Neural Multi-Task Logistic Regression; DeepSurv, Non-Linear Cox Proportional Hazards Deep Neural Networkl; NRI, Net Reclassification Improvement; LR, Logistic Regression; TB, Treebag; Adaboost, Adaptive Boosting; ANN, Artificial Neural Network; LASSO-Cox, Least Absolute Shrinkage and Selection Operator-Cox; PR Curve, Precision-Recall curve.

**Figure S1. Methodology Flow Chart of Data Analysis**


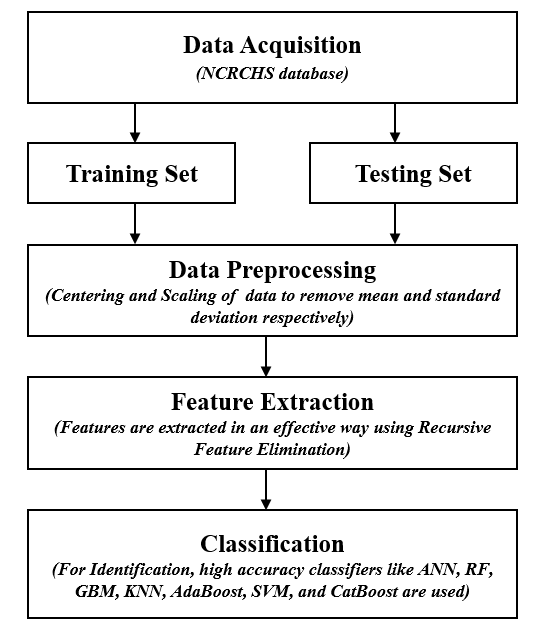

Supplement: Supplementary file 2 — Additional file 2: Table S1. List of Candidate Variables Used in this Study. Table S2. Results of Hyper-parameter Tuning with Different Algorithms in ML-based ASCVD – Models. Table S3. Estimation of Five-year ASCVD Risk Using the PCE (White) and China-PAR Models. Table S4. Comparison with Similar Studies. Figure S1. Methodology Flow Chart of Data Analysis. [file 12911_2023_2242_MOESM2_ESM.docx]
